# Histoepigenetic analysis of HPV- and tobacco-associated head and neck cancer identifies both subtype-specific and common therapeutic targets despite divergent microenvironments

**DOI:** 10.1038/s41388-018-0659-4

**Published:** 2019-01-17

**Authors:** Ivenise Carrero, Hsuan-Chen Liu, Andrew G. Sikora, Aleksandar Milosavljevic

**Affiliations:** 1 Molecular and Human Genetics Department, Baylor College of Medicine, Houston, TX USA; 2 Epigenome Center, Baylor College of Medicine, Houston, TX USA; 3 Translational Biology and Molecular Medicine Program, Baylor College of Medicine, Houston, TX USA; 4 Department of Otolaryngology-Head and Neck Surgery, Baylor College of Medicine, Houston, TX USA; 5 Program in Quantitative and Computational Biosciences, Baylor College of Medicine, Houston, TX USA

**Keywords:** Cancer genomics, Target identification, Cancer microenvironment, Oral cancer

## Abstract

Although head and neck squamous cell carcinoma (HNSCC) has in the past been largely associated with tobacco use, human papillomavirus (HPV+) oropharynx cancer has in recent years emerged as the fastest growing type of HNSCC. Patients with HPV+ HNSCC have a better prognosis; however, the 5-year survival for both HPV+ and HPV− subtypes with recurrent or metastatic disease is poor. To gain insights into the tumor microenvironments of both HNSCC subtypes and identify potential therapeutic targets, we performed epigenomic deconvolution on 580 HNSCC samples from the TCGA dataset. Deconvolution revealed distinct molecular and histoepigenetic profiles of the two tumor subtypes, including their cellular composition, epigenomic profiles and gene expression for constituent cell types, and potential cancer cell-specific targets. Our analyses show that high abundance of both CD8 T-cells and B-cells explains better prognosis in HPV+ HNSCC. Deconvolution of gene expression profiles revealed higher expression of the immunotherapy target PD-1 in HPV+ immune cells compared to HPV− cells, suggesting that HPV+ tumors may preferentially benefit from PD-1 targeted therapy. Further analyses identified HPV+ and HPV− cancer cell surface proteins that can also serve as potential targets for therapy. Specifically, Wnt pathway receptor ROR2 is preferentially overexpressed in HPV+ subtypes, suggesting opportunities for development of targeted therapy based on HPV status. In summary, the comprehensive molecular and histoepigenetic analysis of tumor microenvironments by epigenomic deconvolution reveals potential novel biomarkers and targets for precision therapy of HNSCC.

## Introduction

Head and Neck Squamous Cell Carcinoma (HNSCC) arises from the squamous epithelial cells in the mucosal lining of the oral cavity [1]. The annual worldwide incidence of 550,000 cases makes it the sixth most common cancer [2]. HNSCC can be divided into HPV+ subtype caused by Human Papillomavirus infection, and HPV− subtype that is largely attributable to tobacco and alcohol consumption [3]. While the incidence of HPV− HNSCC is higher worldwide than HPV+, the rate of occurrence of HPV+ is on the rise in the United States [4, 5]. Despite the advancement in new treatments for both subtypes of HNSCC, the 5-year survival rate for head and neck malignancies remains around 65% [6]. While the HPV+ HNSCC patients have a better prognosis and survival [5, 7], the factors that contribute to this difference are still poorly understood.

Targeted therapy has in the past few decades become an established approach for cancer treatment [8]. Monoclonal antibody treatment targeting the epidermal growth factor receptor (EGFR) has been approved for HNSCC, with resistance frequently developing [9]. Immunotherapy targeting PD-1 has been approved for certain subsets of recurrent/refractory HNSCC. However, only a minority of HNSCC patients respond to anti-PD-1 or anti-PD-L1 antibody therapies [10]. The full spectrum of potential targets in HNSCC remains to be identified.

Comprehensive molecular profiling of HPV+ and HPV− HNSCC tumors revealed distinct molecular etiologies, with a high percentage of HPV− tumors carrying TP53 mutations, while a high percentage of HPV+ tumors showing overexpression of p16INK4a [11, 12]. Most recently it was shown that HPV infection not only affects gene expression patterns in HNSCC, but also DNA methylation patterns [13, 14]. While the emerging information about molecular differences and commonalities between the two tumor types suggests the presence of subtype-specific targets and therapy responses, these differences are yet to be fully mapped and translated into precision therapies that are informed by HPV status.

To help develop precision therapies for HPV+ and HPV− HNSCC and to elucidate the factors that affect their prognosis, we set out to identify differences and similarities in HPV+ and HPV− HNSCC tumors at the molecular, cellular and microenvironment levels. We also identify potential new biomarkers or therapy targets. One of our target groups are cell surface proteins, which represent a group of genes widely used to develop targeted therapies [15–17] and immunotherapy treatments [18, 19].

Identification of therapy targets in tumors represents a challenge due to the presence of different cell types in the tumor microenvironment. Previous studies have attempted to look for targets in HPV+ and HPV− HNSCC without taking into consideration the complexity of the cell type composition of tumors [20]. These studies on bulk tumor may lead to both false positives and false negatives as the intercellular differences are confounded by differences in cellular composition. Physical separation methods such as laser capture microdissection and cell sorting can isolate the different cell types in the tumor, however, their throughput is limited [21]. Attempts to address the problem computationally include in silico deconvolution using gene expression or epigenomic (DNA methylation) profiles. The main obstacle in applying current gene expression-based deconvolution methods is the lack of highly accurate gene markers for cells other than leukocytes [22–24]. Some epigenomic deconvolution methods detect only subsets of cell types present within the tumor tissue [25, 26]. To address these problems, we apply the recently developed epigenomic deconvolution (EDec) method [27] to the HNSCC dataset from The Cancer Genome Atlas (TCGA) to comprehensively estimate the histoepigenetic profiles of tumors, including cell type proportions, methylation profiles and gene expression profiles of constituent cell types in both HPV+ and HPV− HNSCC.

## Results

### Epigenomic deconvolution identifies constituent cell types and their methylation profiles

**Fig. 1:**
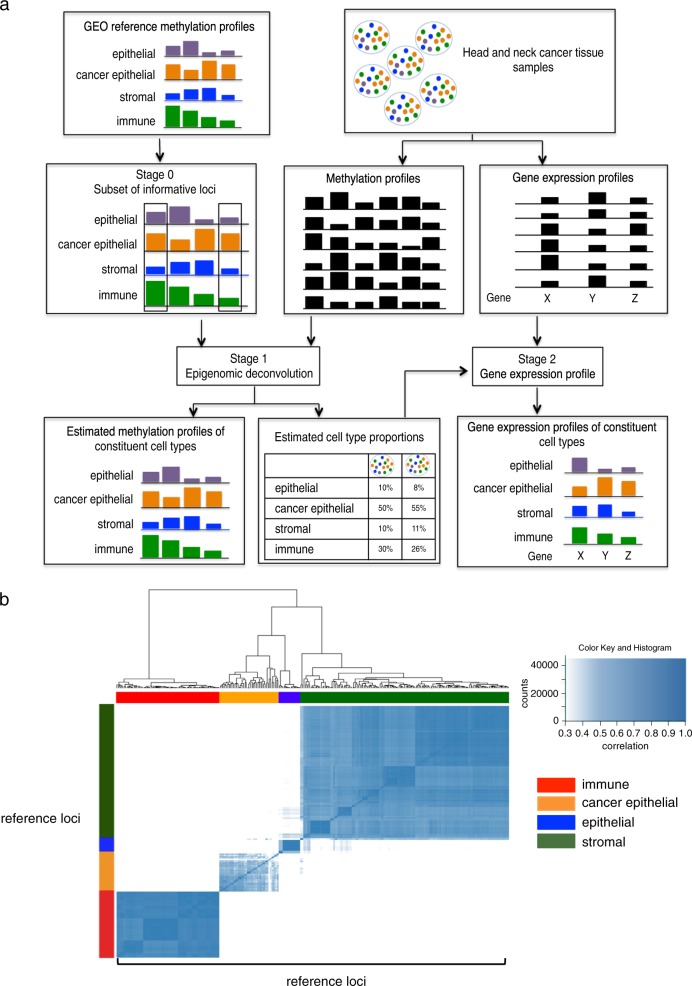
**a** EDec consist of three stages (stages 0, 1, and 2). In stage 0, a set of reference DNA methylation profiles (in our case from the GEO database) is used to select a set of informative loci that show methylation level differences between the cell types expected to be observed in the tumor. In stage 1, the tumor DNA methylation profiles (in our case the HNSCC portion of TCGA) are used to estimate both the average methylation profiles of constituent cell types across tumor samples and cell type proportions in each sample. In stage 2, the estimated cell type proportions as well as the gene expression profiles from the same set of samples (HNSCC portion of TCGA) are used to estimate the gene expression profiles of constituent cell types. **b** Heat map representing the high correlation of the informative reference loci from stage 0 with the constituent cell types from the reference methylation profiles

**Fig. 2:**
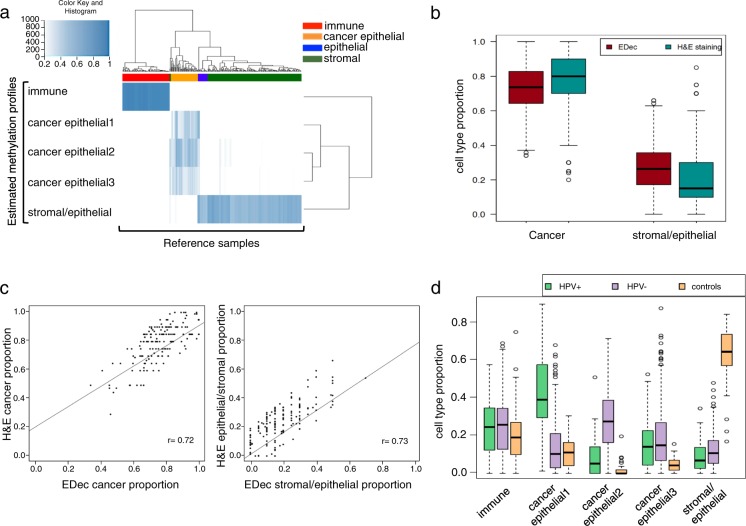
**a** Heat map showing the correlation between the head and neck methylation profiles estimated by EDec and the reference methylation profiles. **b** Boxplot comparing the cell type proportion of cancer cells and stromal/epithelial cells between EDec and H&E staining. **c** Scatterplot showing the correlation between EDec and H&E staining for cancer cells and stromal/epithelial cells. **d** Proportions of constituent cell types for TCGA HNSCC dataset estimated by EDec, comparing HPV+, HPV− and controls

Stage 1 of the EDec method (Fig. 1a) revealed five constituent cell types, their epigenomic profiles, and provided estimates of proportions of each of the five cell types in each tumor sample. To establish the identity of the constituent cell types their estimated methylation profiles were correlated with the reference methylation profiles of known cell types from GEO (Fig. 1b). High correlation suggested that one of the profiles belongs to an immune cell type, one to normal epithelial/stromal and three to distinct cancer epithelial cell types (Fig. 2a).

The estimated proportions of constituent cell-types in each tumor sample were next validated by H&E staining data from the HNSCC component of the cancer digital slide archive (CDSA) from Emory University. CDSA includes 255 HNSCC samples from the TCGA collection and provides proportions of stromal, epithelial, and cancer cells for each tumor biopsy [28]. Since the cell type proportion of stromal and normal epithelial could not be stably deconvoluted, for the purpose of comparison, we combined their CDSA proportion estimates. Average cell proportions estimated by EDec and H&E staining were similar: the average cancer cell proportion as estimated by EDec is 0.73 vs 0.77 by H&E staining; and the stromal/normal epithelial cell proportion estimated by EDec was 0.26 vs 0.21 by H&E staining (Fig. 2b). Sample-to-sample proportion correlation between EDec and H&E was high for a majority of samples, with, 62% of the samples showing correlation of cancer cell proportions at *r* = 0.72 level (*p*-value = 4.06E−26) and stromal/normal epithelial proportions at *r* = 0.73 level (*p*-value = 2.42E−27) (Fig. 2c). This level of correlation validates that EDec performed as expected on this dataset.

### Deconvolution reveals significant epigenomic differences between HPV+ and HPV− cancer cells

Analysis of cell type proportions for 72 HPV+ HNSCC, 243 HPV− HNSCC and 50 adjacent normal samples shows that, as expected, the 50 normal samples have higher proportion of stromal/epithelial cells and negligible proportions of cancer cells compared to the tumor samples. Deconvolution models the cancer fraction by three distinct cancer cell types, one corresponding to HPV+ HNSCC, another to HPV− HNSCC and one present in both (Fig. 2d). The dendrogram of the three deconvoluted cancer cell methylation profiles (Fig. 2a) shows that the HPV+ methylation profile clusters apart from the other two, suggesting highly distinct epigenetic properties of the HPV+ cancer cell type compared to all other HPV− cancer epithelial cells, consistent with previous findings that HPV+ is biologically and epigenetically different from HPV− HNSCC [13, 29].

To further explore the epigenetic differences between the HPV+ and HPV− subtypes, we performed differential methylation analysis. We first identified differentially methylated genes using the RnBeads package [30] and then performed gene set enrichment analysis using GSEA [31, 32]. A number of pathways were specifically upregulated and downregulated in HPV+ HNSCC (Supplementary Table 1a–d). Genes regulated by the transcription factors NANOG and MYC were significantly enriched in promoters hypomethylated in HPV+ HNSCC, suggesting their activation in this subtype (Supplementary Table 1d). This is consistent with the fact that activation of NANOG and MYC targeted genes is associated with poorly differentiated tumors [33], and the poorly differentiated histology of HPV+ HNSCC tumors [3]. In addition, we observed enrichment of genes involved in cell cycle regulation. In contrast, the genes known to be downregulated in nasopharyngeal carcinoma showed gene body hypomethylation, consistent with their silencing (Table 1, supplementary Table 1b).

**Table 1:**
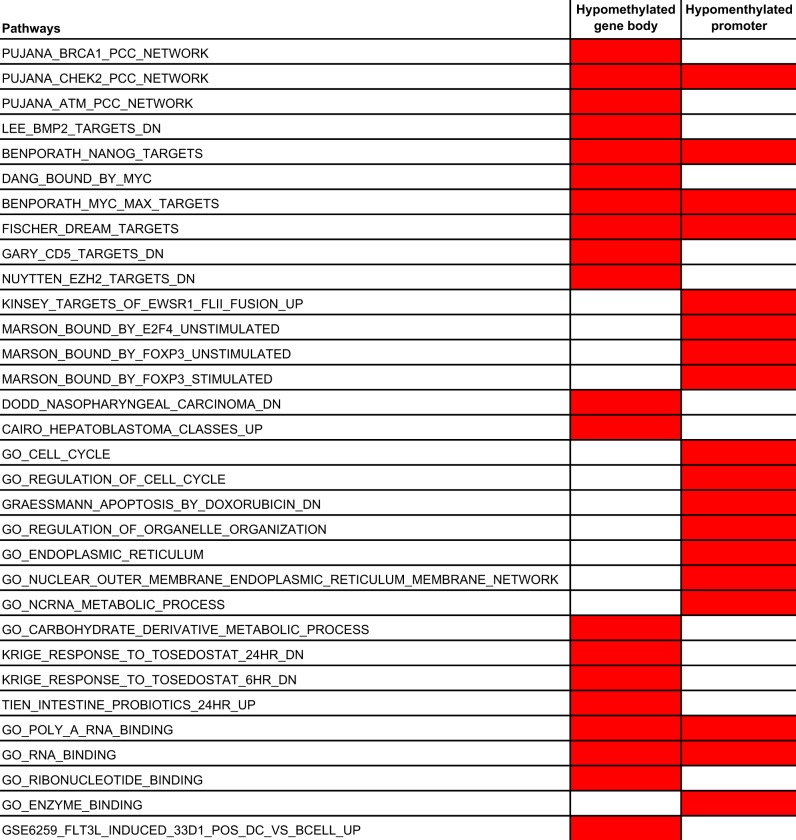
Enriched pathways in hypomethylated promoters and gene bodies

The set of genes with hypermethylated promoters showed enrichment for pathways involved in cancer, such as receptor signaling pathways and histone modifications. We also observed enrichment for genes targeted by the Polycomb proteins SUZ12 and EED, which are known to be repressed in histologically poorly differentiated tumors [33] (Supplementary Table 1c). The set of genes with hypermethylated gene bodies showed enrichment for pathways involved in cancer, including histone modification, cell adhesion, and cell development. Another set of enriched genes were RB1 targets, consistent with the mechanism of carcinogenesis of HPV, where the oncogenic HPV protein E7 repress the Retinoblastoma protein (Rb) and promotes cell proliferation [34] (Supplementary Table 1a). We also observed enrichment of genes related to immune components including CD8 T-cells, suggesting interaction between cancer cells and immune cells in HPV+ HNSCC (Table 2).

**Table 2:**
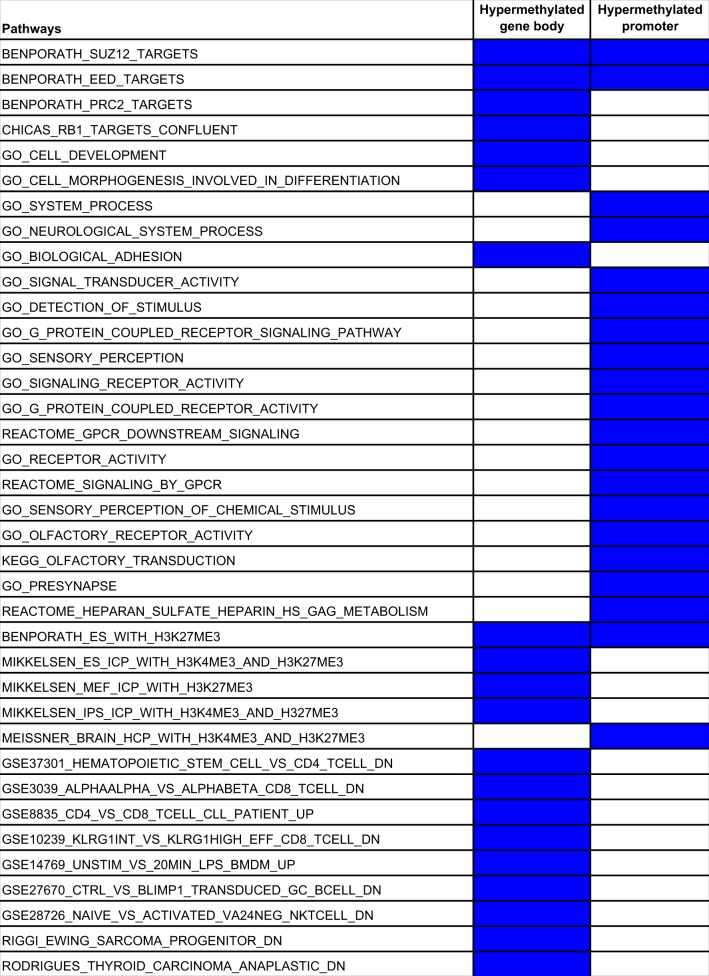
Enriched pathways in hypermethylated promoters and gene bodies

### Higher proportion of CD8+ T-cells and B-cells accounts for better prognosis in HPV+ HNSCC

**Fig. 3:**
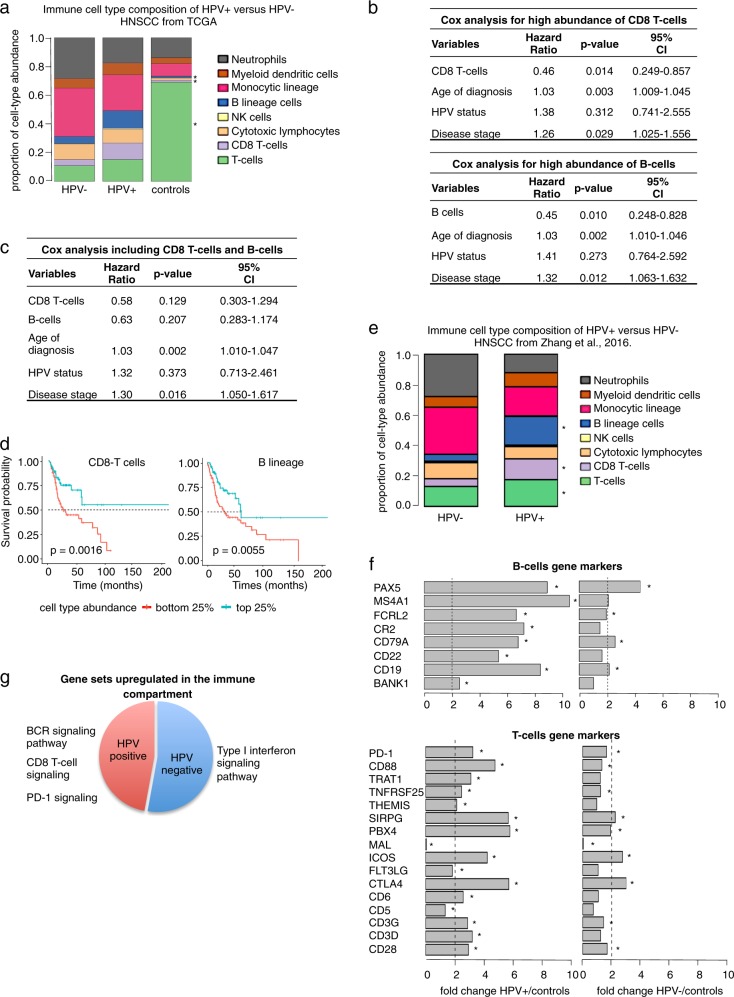
**a** Bar plot representing the immune cell type composition of HPV+, HPV− HNSCC, and controls. **b** Multivariable Cox regression for CD8 T-cells and B lineage indicating their significant independent prognostic values after correction of covariates. **c** Multivariable Cox regression analysis showing that CD8 T-cell and B-cell abundance are not independent prognostic factors. **d** Kaplan–Meier plot showing the significant difference in prognosis for high vs low abundance of CD8 T-cells and B lineage. **e** Bar plot showing the immune cell-type composition with an independent RNA-seq dataset. **f** Bar plot showing the fold change of expression of T-cells and B-cells gene markers in HPV+ and HPV− HNSCC. **g** Summary of gene set enrichment analysis for upregulated immune genes in HPV+ and HPV− HNSCC

Previous studies have shown that HPV+ has higher T-cell infiltration than HPV− HNSCC, however they have not compared tumor profiles with those of normal samples [10]. Our analyses show that both HPV+ and HPV− HNSCC show higher proportion of immune cell types compared to healthy tissue (Fig. 2d). To examine differences in relative proportions of immune cell types between HPV+, HPV− HNSCC, and normal tissue in more detail, we applied the in silico tool MCP counter [35]. Compared to normal samples, both HPV+ and HPV− in fact show relative depletion of T-cells, consistent with the known immunosuppressive microenvironment of HNSCC [36]. However, HPV+ tumors showed significantly higher abundance of CD8 T-cells compared to not only HPV− but also to normal samples, consistent with previous findings [10]. Moreover, HPV+ HNSCC also showed high abundance of B-lineage cells compared to both HPV− and normal samples (Fig. 3a).

Consistent with previous studies [3, 37], Kaplan–Meier analysis showed better survival for HPV+ compared to HPV− HNSCC patients (Supplementary Fig. 1a). However, using multivariate Cox regression we discovered that HPV status loses significance after adjusting for the abundance of CD8 T-cells and/or B-cells along with other factors. We then analyzed the prognostic impact of the abundance of CD8 T-cells and B-cells by controlling for HPV status, age of diagnosis and cancer stage using multivariate Cox regression. In the presence of those covariates, tumors with high abundance of CD8 T-cells and B-lineage cells are associated with increased survival (HR = 0.46, *p*-value = 0.014, 95% CI 0.249–0.857, and HR = 0.45, *p*-value = 0.010, 95% CI 0.248–0.828, respectively) (Fig. 3b, Supplementary Table 2). Interestingly, multivariate Cox regression performed considering CD8 T-cells and B-cell abundances together suggests that these two variables do not provide information about survival independently of each other (Fig. 3c) and are thus largely interchangeable as survival indicators.

To further explore the relation between HPV status and the abundance of CD8 T-cells and B-cells we applied generalized linear modeling. We observed that HPV status is significantly associated with both CD8 T-cell and B-cell abundance (*p*-value = 1.82E−01 and *p*-value = 0.01, respectively) (Supplementary Fig. 1b). Additionally, we examined the collinearity between HPV status, CD8 T-cell, and B-cell abundance by calculating the variance inflation factor (VIF) in our generalized linear model. We observed that both B-cells and CD8 T-cells showed low collinearity (VIF = 1.0829 and VIF = 1.0828, respectively), suggesting that our model is stable. To further explore the relationship between high abundance of CD8 T-cells and high abundance of B-cells, we also performed pairwise correlation between the two variables. We observed an imperfect but significant positive linear correlation between the abundance of CD8 T-cells and B-cells (Pearson’s *r* = 0.27, *p*-value = 1.675E−06, 95% CI = 0.16099–0.3674) (Supplementary Fig. 1c).

To further establish whether immune compartment differences between HPV+ and HPV− HNSCC account for the differences in prognosis between the two subtypes, we compared survival of TCGA patients in the top and bottom immune cell type abundance quartiles using Kaplan–Meier analysis. Consistent with previous studies, patients with high (top 25%) abundance of CD8− T-cells showed better survival than patients with low (bottom 25%) abundance of CD8 T-cells (*p*-value = 0.0016). Moreover, we observed the same survival pattern when comparing tumors with high vs low abundance of B-lineage cells (*p*-value = 0.0055) (Fig. 3d).

Since HPV+ HNSCC is more common in lymphoid-rich regions (oropharyngeal, tonsils, and base of tongue), we asked if the abundance of CD8 T-cells and B-cells in HPV+ HNSCC might be a result of confounding due to the tendency of HPV+ HNSCC to occur in those regions. Toward this goal, we compared the immune cell-type proportion differences between HPV+ and HPV− HNSCC tumors present only in these lymphoid-rich regions. HPV+ HNSCC tumors showed a significantly higher abundance of CD8 T-cells and B-cells compared to HPV− HNSCC (Supplementary Fig. 1d), suggesting that the higher abundance of CD8 T-cells and B-cells in HPV+ HNSCC cannot be explained by their preferential localization in lymphoid-rich regions.

To further corroborate the higher abundance of CD8 T-cells and B-cells in HPV+ compared to HPV− HNSCC, we used an independent dataset from Zhang et al. (2016) [38], consisting of 18 HPV+ and 18 HPV− RNA-seq profiles from pre-treated HNSCC. Consistent with our analyses of TCGA head and neck cancer data, MCP counter analysis of HPV+ tumors showed a significantly higher abundance of CD8 T-cells and B-cells compared to HPV− HNSCC (Fig. 3e).

### Expression of PD-1 and CTLA4 in the immune compartment may predict differential and shared response to immunotherapy in HPV+ and HPV− HNSCC

We next compared the gene expression profiles (deconvoluted by EDec) of the immune fraction in HPV+ and HPV− HNSCC. As expected, marker immune genes for T-cells and B-cells were more highly expressed in HPV+ HNSCC tumors compared to HPV− tumors (Fig. 3f). Interestingly, CTLA4 was highly expressed in both HPV+ (fold change = 3.99, *p*-value = 2.98E–07) and HPV− HNSCC (fold change = 2.5, *p*-value = 0.004). In contrast, PD-1 (PDCD1) is significantly overexpressed only in HPV+ tumors (fold change = 3.21, *p*-value = 0.006). This finding is interesting in light of the clinical trials targeting PD-1 in HNSCC (regardless of HPV status) that show that only a subset of patients show response [10]. Our results are also consistent with the improved response of HPV+ HNSCC tumors to anti-PD-1 therapy observed in some clinical trials [39]. In contrast, our results suggest that immunotherapy targeting CTLA-4 might show comparable response for both subtypes.

### Gene set enrichment analysis corroborates higher CD8+ T-cell and B-cell infiltration and PD-1 overexpression in HPV+ tumors

To gain further insights into the immunological differences between HPV+ and HPV− tumors, we next compared the gene expression profiles in the immune compartment estimated by EDec between HPV+, HPV− and normal samples. We performed gene set enrichment analysis [31] on the immune gene set differentially expressed in HPV+ and HPV− HNSCC (Supplementary Table 3a–c). Consistent with the high abundance of CD8− T cells in HPV+ HNSCC, we observed enrichment of pathways involved in CD8 T-cell signaling. Moreover, consistent with high abundance of B cells, we observed enrichment of pathways for B-cell receptor (BCR) signaling. PD-1 signaling pathways were enriched in HPV+ HNSCC, consistent with the expression of PD-1 itself. In the case of HPV−, we observed enrichment for regulation and cellular response to type I interferon (Fig. 3g, Supplementary Table 4a–c).

### Deconvolution analysis reveals overexpressed cell surface proteins in HNSCC cancer cells

**Fig. 4:**
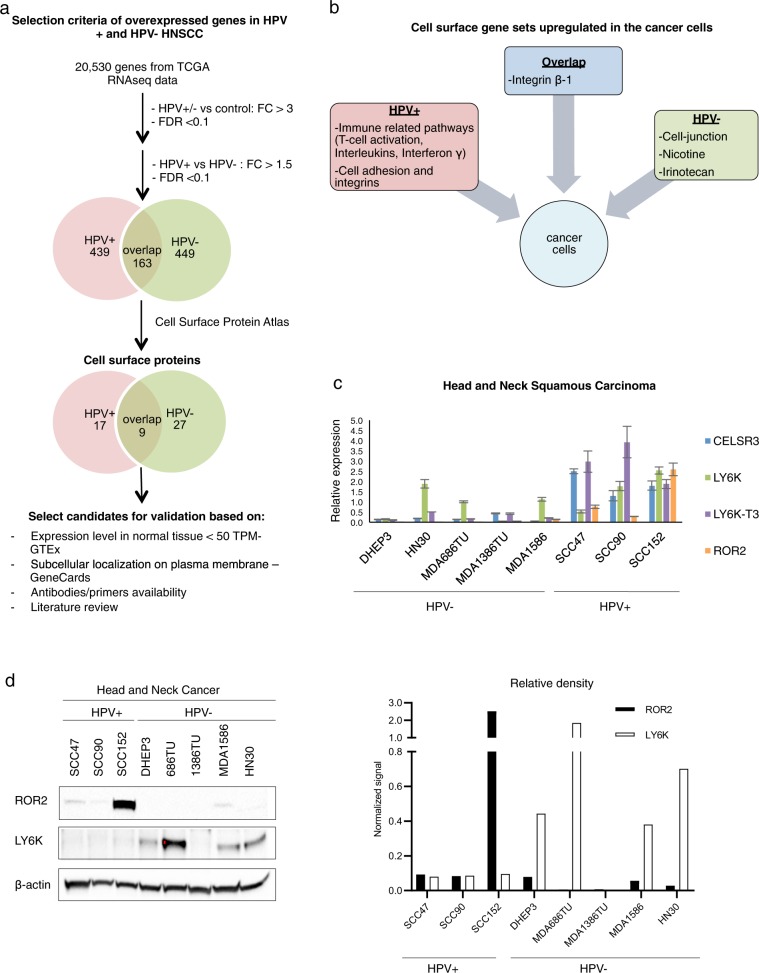
**a** Workflow showing the selection criteria of cell surface candidates and Venn diagram showing the number of genes overexpressed in cancer cells in HPV+ and HPV− HNSCC. **b** Summary of gene set enrichment analysis of cell surface genes upregulated in HPV+, HPV− and overlapping between both subtypes. **c** Bar plot showing qRT-PCR analysis of candidate cell surface genes in HPV+ and HPV− HNSCC cell lines. **d** Western blot analysis and the bar graph of relative density after corrected to β-actin showing expression of ROR2 and LY6K in HPV+ and HPV− HNSCC cell lines

Because of their accessibility, cancer cell surface proteins are potential targets for therapy using both antibodies and small molecules. We therefore analyzed the expression of cell surface proteins for HPV+ and HPV− subtypes of HNSCC by estimating the gene expression profiles of cancer cells (EDec stage 2) using the normalized TCGA HNSCC RNA-seq data. Toward this goal, we combined the three cancer epithelial estimated proportions from EDec stage 1. EDec stage 2 was independently applied to 72 HPV+ and 243 HPV− HNSCC tumors and 20 normal tissue controls. EDec predicted overexpression (compared to normal epithelial/stromal cells) of 439 genes in HPV+, 449 in HPV− and 163 genes in both HPV+ and HPV− cancer cells (Fig. 4a and Supplementary Table 5a–c). To identify the cell surface proteins among those that are overexpressed, we compared our set of overexpressed genes with The Cell Surface Protein Atlas [35]. The analysis revealed 17 cell surface proteins overexpressed in HPV+, 27 in HPV− and nine cell surface proteins overexpressed in both subtypes (Fig. 4a, Table 3).

**Table 3:** Cell surface proteins overexpressed in cancer cells

Preferentially overexpressed in HPV+ HNSCC							
genes	mean of expression controls	mean of expression HPV+	fold change HPV+/controls	FDR	mean of expression HPV-	fold change HPV-/controls	FDR
ITGAM	70.80	370.67	5.24	5.24E-04	19.24	0.27	5.11E-01
VCAM1	169.55	4133.39	24.38	8.23E-24	0.00	0.00	2.79E-01
TMEM26	5.16	27.92	5.41	2.20E-06	4.67	0.91	9.34E-01
NTN3	1.55	10.54	6.81	2.27E-13	1.87	1.21	6.75E-01
PDCD1LG2	63.14	259.03	4.10	3.89E-06	58.76	0.93	7.18E-01
PON1	0.90	3.23	3.57	1.15E-03	0.66	0.73	7.24E-01
ADAM22	8.12	58.64	7.22	1.39E-09	12.89	1.59	4.23E-01
SLC12A5	1.51	9.51	6.29	7.36E-14	2.59	1.72	8.83E-02
CRB2	20.66	187.34	9.07	7.13E-18	6.69	0.32	9.55E-02
ITGA2B	8.70	50.16	5.77	5.06E-24	17.67	2.03	4.56E-05
OLFM1	267.08	2961.37	11.09	1.02E-13	758.81	2.84	9.61E-03
ZP4	0.58	3.40	5.82	1.61E-04	1.65	2.82	9.25E-02
CELSR3	118.58	737.21	6.22	2.82E-37	304.07	2.56	3.04E-10
ROR2	94.70	522.15	5.51	1.54E-08	289.49	3.06	6.37E-04
CLEC1A	22.28	124.37	5.58	3.69E-05	72.79	3.27	1.98E-02
ICAM1	578.30	3194.41	5.52	1.09E-02	1384.93	2.39	4.35E-01
PTGIR	15.28	86.87	5.68	5.89E-07	13.63	0.89	9.18E-01

Preferentially overexpressed in HPV- HNSCC							
genes	mean of expression controls	mean of expression HPV-	fold change HPV-/controls	FDR	mean of expression HPV+	fold change HPV+/controls	FDR
ALPP	0.61	31.28	51.34	2.41E-125	0.00	0.00	9.22E-01
NT5E	213.94	1257.01	5.88	1.75E-04	387.26	1.81	5.03E-01
SGCZ	0.26	2.10	8.13	2.25E-02	0.20	0.76	9.76E-01
IL13RA2	21.29	94.73	4.45	4.85E-06	21.58	1.01	1.00E+00
ODZ1	0.22	56.86	254.91	4.79E-06	0.00	0.00	1.00E+00
PTPRD	77.15	283.72	3.68	1.10E-04	40.38	0.52	4.50E-01
BCHE	27.30	111.63	4.09	8.96E-04	3.02	0.11	3.10E-01
ORM1	0.58	8.63	14.95	8.47E-06	0.00	0.00	7.47E-01
NTSR1	9.37	62.44	6.67	1.10E-39	1.78	0.19	3.80E-04
FBN2	212.38	1626.51	7.66	5.89E-15	426.47	2.01	1.80E-02
FGFR4	46.56	200.06	4.30	2.94E-05	107.51	2.31	5.09E-02
HTR1D	2.40	29.31	12.22	5.25E-14	3.83	1.60	3.79E-01
TFPI2	38.36	357.56	9.32	9.91E-13	0.00	0.00	1.54E-01
MDGA1	141.13	490.76	3.48	7.56E-07	143.25	1.02	1.00E+00
NXF2	13.20	67.24	5.09	1.12E-10	0.00	0.00	1.48E-02
MMP13	159.86	4039.67	25.27	7.03E-46	322.68	2.02	6.61E-02
PCDHGB1	33.00	121.46	3.68	5.06E-10	13.96	0.42	2.58E-02
PROCR	175.03	899.66	5.14	2.08E-08	440.34	2.52	3.15E-03
F2	0.30	5.53	18.65	9.83E-47	0.00	0.00	1.33E-01
CDH4	5.15	41.35	8.03	1.53E-04	0.00	0.00	5.65E-01
ITIH2	0.21	1.22	5.91	4.05E-04	0.00	0.00	4.65E-01
TSPAN18	265.29	872.08	3.29	2.50E-03	272.16	1.03	1.00E+00
PTGS2	256.78	2080.61	8.10	4.12E-06	1050.23	4.09	1.41E-02
CLEC12A	0.56	33.17	59.74	6.14E-05	2.11	3.81	8.65E-01
CD70	2.98	90.85	30.52	1.32E-07	50.91	17.11	5.52E-03
SDK1	145.67	1152.86	7.91	2.50E-03	619.05	4.25	1.36E-01
ABCC2	13.09	59.09	4.51	4.73E-04	1.75	0.13	3.59E-01

Overexpressed in both HPV+ and HPV- HNSCC							
genes	mean of expression controls	mean of expression HPV+	fold change HPV+/controls	FDR	mean of expression HPV-	fold change HPV-/controls	FDR
CHST7	60.94	213.32	3.50	4.01E-09	289.09	4.74	2.77E-11
TMEM200B	55.56	177.05	3.19	5.43E-06	184.94	3.33	2.78E-06
NXPH4	271.45	910.32	3.35	3.39E-03	878.77	3.24	4.89E-03
COL11A1	32.91	282.92	8.60	3.56E-02	235.69	7.16	7.99E-02
MGAT5B	5.92	44.42	7.50	5.46E-07	35.85	6.05	2.23E-05
LY6K	434.86	1471.16	3.38	1.27E-14	1458.68	3.35	1.24E-11
ICAM5	20.12	109.12	5.42	2.65E-16	101.61	5.05	1.14E-11
EPHB2	132.77	537.62	4.05	7.53E-05	594.52	4.48	1.64E-05
PLAU	805.67	3685.90	4.57	2.04E-07	5178.89	6.43	7.24E-10

### Gene set enrichment analysis of cell surface proteins suggests cross-talk between cancer and immune cells

We next performed gene set enrichment analysis on the cell surface genes preferentially overexpressed in HPV+ or HPV− or both HNSCC subtypes. Cell surface genes in HPV− tumors were enriched for Nicotine pathway, consistent with the role of tobacco consumption in HPV− HNSCC. Another enriched pathway was cell–cell junction, implicated in the process of epithelial to mesenchymal transition (EMT), leading to invasion and metastasis [40]. Interestingly, we observed enrichment for pathways involving the drug Irinotecan, which has been tested in a clinical trial for HNSCC [41]. Though the trial failed, the fact that Irinotecan pathway activation is observed in HPV− tumors but not in HPV+ tumors suggests that Irinotecan therapy response might vary by HPV status (Fig. 4b).

In HPV+ HNSCC we observed enrichment of cell adhesion and integrin pathways, which have previously been implicated in metastasis [42, 43], consistent with the relatively increased frequency of regional and distant metastasis in HPV+ HNSCC. Strikingly, we observed enrichment of immune-related pathways including T-cell activation, interleukin signaling, and interferon γ signaling (Fig. 4b) in HPV+ HNSCC. In fact, one of the overexpressed cell surface proteins, PDCD1LG2 (PD-L2), is known to be overexpressed in the T-cell activation pathway, it can bind to PD-1 and regulates T-cell-mediated immune response playing a role in immune escape [44, 45], consistent with our findings of overexpression of PD-1 in HPV+ T-cells. These findings suggest a cross-talk between the cancer cells and T-cells in the tumor microenvironment of HPV+ HNSCC tumors. Among the cell surface genes overexpressed in both subtypes of HNSCC, we observed enrichment for the ß1 integrin pathway, consistent with previous research targeting this pathway in HNSCC [46] (Fig. 4b, Supplementary Table 6a–c).

### Prioritization of cancer cell surface proteins for validation experiments

In order to select candidates for validation, we screened the cell surface proteins based on their subcellular localization, expression level in normal tissue according to the GTEx portal from the Broad Institute [47], literature research, and availability of primers/antibodies. The selection process, depicted in Fig. 4a, resulted in the following two types of validation candidates: (1) those highly expressed in both HPV+ and HPV− HNSCC: LY6K, and (2) those preferentially expressed in HPV+ HNSC: ROR2, VCAM1, ICAM1, ITGA2B, PTGIR, and CELSR3. In the following two sections, we discuss validation of candidates in both categories.

### LY6K as a possible common target for HPV+ and HPV− HNSCC

Transcription of the cancer-testis antigen LY6K was validated in several HPV+ and HPV− HNSCC cell lines (Fig. 4c). LY6K-T3 is the cell surface isoform that has a shorter c-terminal region than other isoforms; however, the biological differences between the three isoforms of LY6K are still unknown. The LY6K transcript variant LY6K-T3 was highly expressed in HNSCC HPV+ cell lines, while LY6K transcript variants 1 and 2 were expressed in both HPV+ and HPV− cell lines. We also observed overexpression of LY6K in HPV+ cervical cancer cell lines (Supplementary Fig. 2a).

We next evaluated protein expression of LY6K by Western blot analysis. LY6K showed higher expression in HPV− when compared to HPV+ HNSCC (Fig. 4d) but showed high expression in HPV+ cervical cancer cell lines (Supplementary Fig. 1b). These results suggest that HPV-associated cancers show biological differences depending on the tissue type. The lack of correlation between transcriptome and proteome in HNSCC cell lines might be due to posttranslational modifications or lack of antibodies to discriminate between the different isoforms. Moreover, flow cytometry analyses of HPV+ and HPV− HNSCC cell lines showed a pattern of similar expression of LY6K in both subtypes (Supplementary Fig. 3a), suggesting that LY6K could be a potential target for both subtypes of HNSCC.

### ROR2 is a potential target for HPV+ HNSCC

Expression of candidates CELSR3 and ROR2 was examined using qRT-PCR in HNSCC cell lines listed in Table 4. We have also examined the expression of these candidate genes in cervical cancer cell lines. We observed overexpression of ROR2 transcripts in HPV+ HNSCC cell lines and absence of expression in HPV− cell lines (Fig. 4c). Interestingly, we observed overexpression of ROR2 in both HPV+ and HPV− cervical cancer cell lines (Supplementary Fig. 2a).

**Table 4:** Cancer cell lines

Cell line	Cancer type	HPV status (+/−)
DHEP3	Head and neck	−
MDA1386TU	Head and neck	−
MDA1586	Head and neck	−
HN30	Head and neck	−
MDA686TU	Head and neck	−
UM-SCC-47	Head and neck	+
UPCI:SCC090	Head and neck	+
UPCI:SCC152	Head and neck	+
UPCI:WCC154	Head and neck	+
c-33a	Cervical	−
SiHa	Cervical	+
Caski	Cervical	+

We could not validate protein expression of ICAM-1, VCAM-1, and PTGIR (Supplementary Fig. 3b) using available reagents. These results may also suggest that cell lines might not fully recapitulate protein expression in tumor tissue, possibly due to loss of the cellular microenvironment upon passaging of cancer cells as a monoculture in the 2-D culture system.

Among the list of candidates examined by Western blot, only ROR2 showed higher protein expression in HPV+ HNSCC than in HPV− HNSCC (Fig. 4d). Moreover, ROR2 also showed higher expression in HPV+ cervical cancer cell lines compared to HPV− cervical cancer cell lines (Supplementary Fig. 2b). Although CELSR3 could not be validated by Western blot, flow cytometry analyses showed a trend towards higher expression in HPV+ HNSCC cells (Supplementary Fig. 3a). Taken together, our RNA and protein analyses suggest that ROR2 is preferentially expressed in HPV+ HNSCC and therefore may potentially serve as a target for HPV+ HNSCC-specific therapy.

## Discussion

To gain insights into the tumor microenvironments of the HPV+ and HPV− HNSCC tumor subtypes and identify potential therapy targets, we performed histoepigenetic analysis of 580 HNSCC tumors from the TCGA dataset. Epigenomic deconvolution revealed their constituent cell types, and potential cancer cell-specific targets. One limitation of our current analyses is the inability to deconvolute normal epithelial from stromal cells, possibly due to high heterogeneity of HNSCC tumors [48].

We identified differences between the epigenomes of HPV+ and HPV− cancer cells and also differences in their corresponding microenvironments. By combining the EDec method of epigenomic deconvolution with gene expression-based MCP Counter analysis, we were able to obtain a more complete assessment of the immune compartment. We observed high abundance of both CD8 T-cells and of B-cells, consistent with previous studies suggesting that B-cells play a role in the priming of CD8 T-cells for activation of immune response [49]. Overall, the immune composition analysis suggests that HPV+ tumors are more immunogenic than HPV− tumors and that CD8 T-cell and B-cell abundance accounts for better survival in HPV+ HNSCC.

We observed enrichment of Type I interferon pathway in HPV− tumors, which plays a double role in cancer, providing signals that help detect and control cancer cells but also in some cases suppressing immune response [50]. These observations suggest that the immune infiltration in HPV− tumors may be predominantly suppressive, while in HPV+ tumors the infiltration includes both cell types associated with relatively better prognosis (i.e., CD8+ T -cells) as well as cell types of uncertain significance (i.e., B c-ells).

Cell-type-specific transcriptome analysis reveals overexpression of PD-1 and PD-L2, a ligand of PD-1 in HPV+ but not in HPV− tumors. In combination with previous findings that detection of both PD-L1 and PD-L2 predict positive response to the drug Pembrolizumab [44], our results suggest that HPV+ tumors may be more responsive to therapies targeting PD-1 and its ligands. In contrast, CTLA4 is highly expressed in both subtypes of cancer, suggesting that therapies targeting CTLA4 and its ligands may be beneficial for both subtypes.

We also identified cell surface proteins in cancer cells that may potentially serve as targets for the development of new therapies. Some of these cell surface targets are expressed in both subtypes and some are subtype-specific. Our results implicate LY6K in both HPV+ and HPV− subtypes. LY6K is known to play a role in several cancers [51, 52], including HPV-associated cervical cancer [53]. LY6K is a highly specific target as it is a cancer-testis antigen expressed exclusively in normal reproductive tissues and also in some cancer cells. While the overexpression of LY6K has been previously reported in HNSCC [54], we observe for the first time that overexpression of LY6K transcript 3 correlates with the HPV status. Further studies will be required to elucidate the function of LY6K transcript 3 in HPV+ HNSCC.

Another potential target is ROR2, a receptor of the non-canonical Wnt pathway that is expressed specifically in HPV+ tumors. Aberrant expression of this pathway has been observed in several cancers [55–57], including tongue squamous cell carcinoma [58]. ROR2 plays a dual role in cancer, as either tumor suppressor or activator depending on the affected tissue [59]. Our observed association of ROR2 with HPV+ status suggests a potential role for non-canonical Wnt pathway in HNSCC, and suggests targeting of ROR2 as a potential therapeutic strategy for HPV+ HNSCC. Such targeted therapies with fewer side effects than cytotoxic chemotherapy are particularly relevant for HPV+ HNSCC because of better prognosis and higher importance of quality of life preservation for patients with this tumor subtype.

In conclusion, histoepigenetic analysis of HNSCC revealed differences and commonalities between HPV+ and HPV− subtypes at the molecular, cellular and tissue levels, providing insights into HNSCC biology and information to guide the development of immunotherapy and other precision therapies for HNSCC.

## Materials and methods

### EDec method

To perform deconvolution of HNSCC, we used the previously described EDec method [27]. In brief, EDec is an in silico deconvolution method that estimates cell type composition of tumors and gene expression profiles for the predicted cell types.

EDec stage 1 estimates constituent cell type proportions and methylation profiles of the constituent cell types. EDec stage 2 estimates the gene expression profiles of constituent cell types (Fig. 1a, stage 2) [27].

### Reference methylation profiles

EDec requires a list of loci that are differentially methylated between constituent cell types and thus informative for deconvolution. To obtain the differentially methylated loci that differentiate epithelial, stromal, immune, and cancer epithelial cells, the DNA methylation profiles from previously published datasets were gathered from NCBI GEO database. Our reference 450k array profile DNA methylation dataset includes 273 samples from 10 different studies (Supplementary Table 7).

To identify the informative loci, we applied EDec stage 0 to the reference profiles, using a *p*-value of 10^−10^. The final reference methylation set contains 400 informative loci (Fig. 1b).

### TCGA datasets

The TCGA methylation and expression data were downloaded from the University of Santa Cruz cancer browser – version 2015.

The 580 DNA methylation profiles were generated using Illumina’s Infinium Human Methylation 450k arrays. The 564 normalized RNA-seq v2 profiles were generated by IlluminaHiSeq. HPV status for 72 HPV+ and 243 HPV− samples was obtained from ref. [60].

### Selecting the number of cell types for deconvolution

In order to select the number of cell types for the deconvolution, we applied a stability criterion. Specifically, EDec stage 1 is run with a random subset of 80% of the TCGA DNA methylation profiles using various numbers of cell types (from 3 to 10). We observed that a five cell-type model showed the best reproducibility of methylation and proportion estimates.

### Gene set enrichment analysis of methylation profiles of the cancer cell types estimated by EDec

Gene set enrichment analysis was performed using GSEA [30]. To perform this analysis, using RnBeads, we selected the top 200 genes with hypermethylated and hypomethylated gene bodies and promoters when comparing HPV+ to HPV− tumors. Using GSEA, we selected the top 20 GSEA gene set enrichments with an FDR <0.05 for each of the four gene sets identified by RnBeads.

### Gene expression profiles of constituent cell types

To estimate gene expression profiles of constituent cell types, we applied EDec stage 2 to the TCGA RNA-seq profiles and cell type proportions (estimated in stage 1). We applied the method independently to the 72 HPV+, 243 HPV− and 20 normal samples that have both DNA methylation data and RNA-seq profiles. To identify genes that are preferentially overexpressed in HPV+ and in HPV−, we performed *t*-tests comparing the means of expression in HPV+ vs normal samples, and HPV− vs normal samples. The threshold to determine differential expression was FDR < 0.1, fold change of gene expression cancer vs normal samples >3. To identify subtype-specific overexpressed proteins we performed a *t*-test comparing the means of gene expression in HPV+ vs HPV−, using a fold change of gene expression >1.5 and FDR < 0.1.

To identify the cell surface proteins, we downloaded the Cell Surface Atlas [61]. Gene set enrichment analysis was performed using consensusPathDB [62] on overexpressed cell surface proteins between HPV+ and HPV− HNSCC.

### Deconvolution of constituent cell types of the immune compartment

To determine the abundance of constituent cell types of the immune compartment, we applied the previously described method MCP counter [35] to the TCGA RNA-seq data. To determine that an immune cell type is differentially abundant (*p*-value < 0.05) and an immune gene is differentially expressed (fold change >2, FDR < 0.1) between HPV+ and HPV−, *t*-test was performed between HPV+ vs normal samples, HPV− vs normal samples. To detect subtype specific overexpressed genes and subtype-specific immune cell-type abundance, a *t*-test was performed between HPV+ vs HPV−.

Kaplan–Meier and Cox regression survival analyses were performed in R using the packages ‘survminer’ and ‘survival’. Gene set enrichment analysis was performed using GSEA [30] on the genes differentially expressed between HPV+ and HPV− HNSCC.

### Statistical analyses

All our statistical analyses were performed using the R programming language and Bioconductor packages. The pairwise correlation analyses were performed with the two sided, Pearson method with a confidence level of 0.95. The Kaplan–Meier survival analyses were performed with the R package ‘survminer’, using the top and bottom 25% quartiles of CD8 T-cells and B-cells abundances. The multivariate Cox regression was performed with the R package ‘survival’, using the top and bottom 25% quartiles of abundance for CD8 T-cells and B-cells, we also included as factors the following values: HPV status, age of diagnosis, and cancer stage. The default method ‘Efron’ was used. The collinearity test was performed with the R package ‘car’ to calculate the variance inflation factors (VIF). The generalized linear model was performed using the family binomial (logit).

### Validation of transcription of target genes using cancer cell lines

The HPV− HNSCC cell lines DHEP3 (gift from Dr. Julio A. Aurirre-Ghiso, Icahn School of Medicine at Mount Sinai), MDA1386TU, MDA1586, HN30, MDA686TU (gift from Dr. Jeffery N. Myers, MD Anderson Cancer Center) were grown in DMEM (Sigma-Aldrich), 10% fetal bovine serum, 100 U/ml penicillin, 100 µg/ml streptamycin, 1% non-essential amino acids, 1% sodium pyruvate and vitamin. The HPV+ HNSCC cell lines UM-SCC47, UPCI:SCC090, UPCI:SCC154, and UPCI:SCC152 (gift from Dr. Susanne M. Gollin, University of Pittsburgh) are cultured in MEM with 10% FBS, non-essential amino acids and gentamicin. They were maintained at 37 °C with 5% CO_2_ in humidified incubator.

Total RNA was extracted using TRIzol reagent (Invitrogen) and the concentration and the purity of RNA were measured by Cytation 3 (BioTek). cDNA was synthesized from 1 µg total RNA using qScript cDNA supermix (Quanta). The gene-specific PCR products were generated with PerfeCta SYBR Green FastMix (Quanta) and reactions carried out in a CFX96 real-time PCR machine (Bio-Rad). Primer sets for the candidate genes are listed in Supplementary Table 8.

### Evaluation of protein expression

Cell pellets were washed with ice-cold PBS twice then scraped from the T75 flask. Cell pellets were lysed in RIPA buffer (Biosciences), phenylmethanesulfonyl fluoride (PMSF), and cocktail inhibitor 2 (Sigma). Then the proteins (30 µg) were resolved by 4–12% SDS-PAGE pre-cast gels (Invitrogen) and subsequently electrophoretically transferred to PVDF membranes. Membranes were incubated with 3% BSA of blocking buffer for 1 h at room temperature then incubated with primary antibodies at 4 °C overnight. Membranes were incubated with corresponding species of secondary antibodies for 1 h at room temperature, followed by the detection with the enhanced chemilumiescence (ECL) system (Thermoscientific Fisher) and visualized by ChemiDoc (Bio-Rad). The relative densities of bands were quantified using ImageJ software (NIH). Sources of antibodies were as follows: anti-LY6K (HPA017770, Sigma-Aldrich), anti-CELSR3 (clone: 763103, R&D Biotechnology), anti-β-actin, anti-ICAM-1, anti-VCAM-1, anti-PTGIR (clone: C4, G5, E10, G7, Santa Cruz Biotechnology), anti-ROR2 (DSHB Hybridoma, University of Iowa).

### Disclaimer

All the authors of this manuscript state the material is original research, has not been previously published and has not been submitted for publication elsewhere while under consideration.

## Supplementary information

Supplementary Figure 1

Supplementary Figure 2

Supplementary Figure 3

Supplementary Table 1a

Supplementary Table 1b

Supplementary Table 1c

Supplementary Table 1d

Supplementary Table 2

Supplementary Table 3a

Supplementary Table 3b

Supplementary Table 3c

Supplementary Table 4a

Supplementary Table 4b

Supplementary Table 4c

Supplementary Table 5a

Supplementary Table 5b

Supplementary Table 5c

Supplementary Table 6a

Supplementary Table 6b

Supplementary Table 6c

Supplementary Table 7

Supplementary Table 8
