# Supplementary figures and images for: Histoepigenetic analysis of HPV- and tobacco-associated head and neck cancer identifies both subtype-specific and common therapeutic targets despite divergent microenvironments
Source: Oncogene. 2019 Jan 17;38(19):3551–68. doi: 10.1038/s41388-018-0659-4 (PMC6756123; doi:10.1038/s41388-018-0659-4)

Supplementary Figure 1

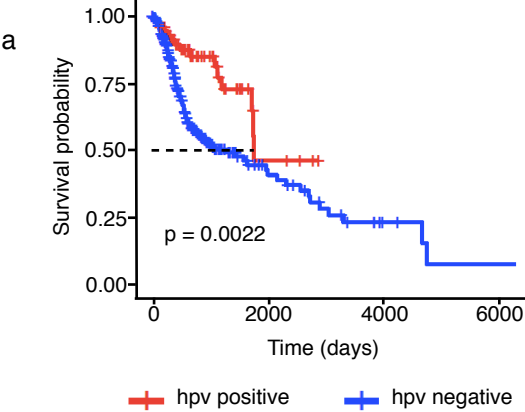

b

| Coefficients | Estimate | p-value  | 95% CI            |
|--------------|----------|----------|-------------------|
| Intercept    | 2.1950   | 2e-16    | 1.7973 - 2.6296   |
| B-cells      | -0.0017  | 0.011    | -0.0031 - -0.0005 |
| CD8 T-cells  | -0.0058  | 1.82e-07 | -0.0081 - -0.0038 |

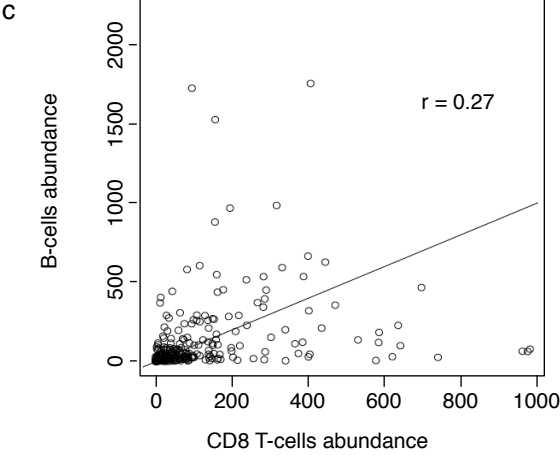

d

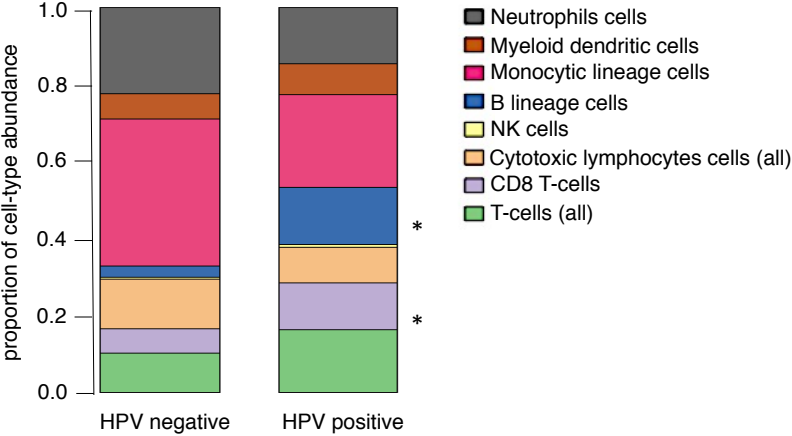

Supplement: Supplementary file 1 — Supplementary Figure 1 [file 41388_2018_659_MOESM1_ESM.pdf]

Supplementary Figure 2

a

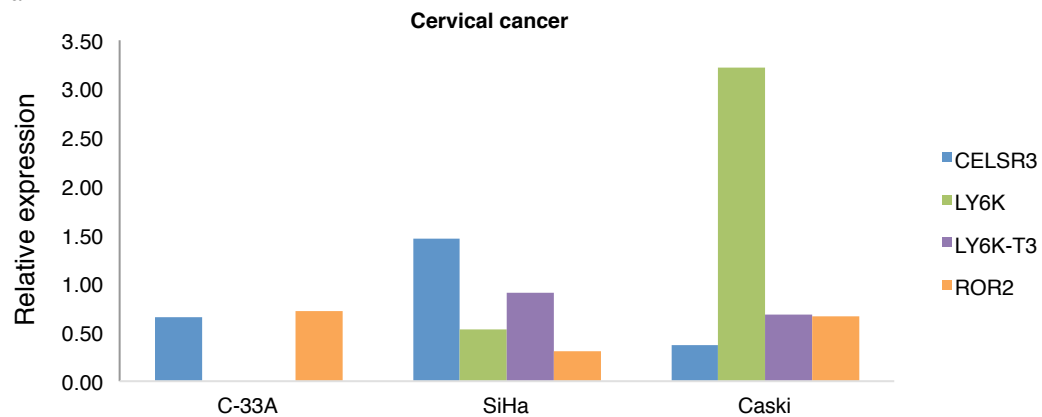

b

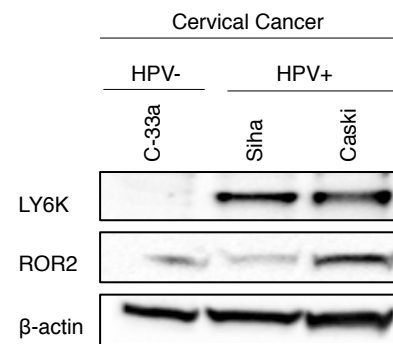

Supplement: Supplementary file 2 — Supplementary Figure 2 [file 41388_2018_659_MOESM2_ESM.pdf]

a

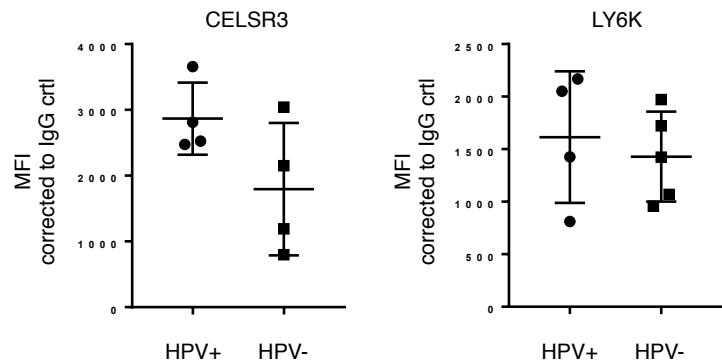

b

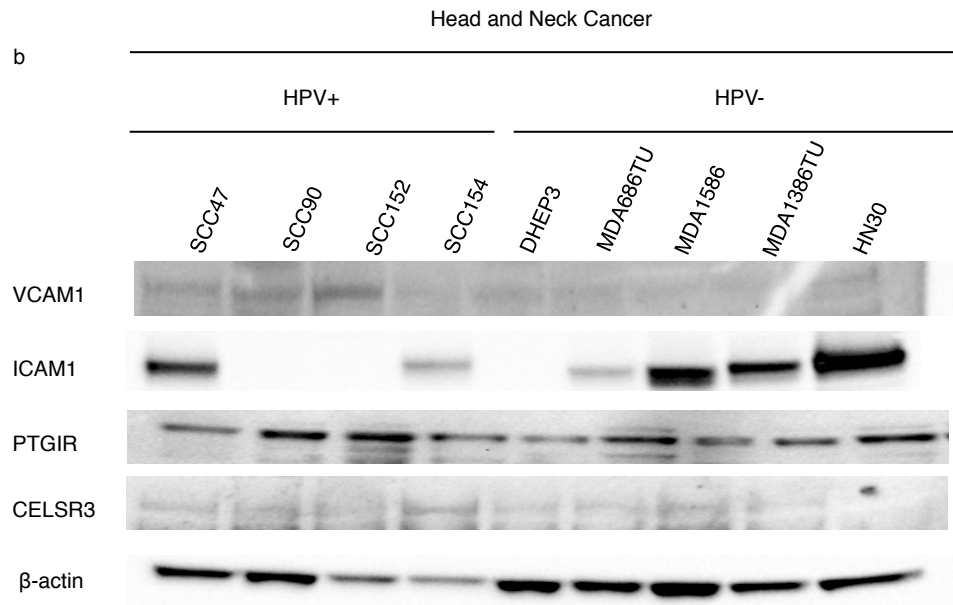

Supplement: Supplementary file 3 — Supplementary Figure 3 [file 41388_2018_659_MOESM3_ESM.pdf]
